# Editorial: Immuno-Epigenetic Markers for Infectious Diseases

**DOI:** 10.3389/fimmu.2019.02719

**Published:** 2019-11-19

**Authors:** Eduardo José Melo dos Santos, Glen N. Barber, Ricardo Ishak, Antonio Carlos Rosário Vallinoto

**Affiliations:** ^1^Human and Medical Genetics Laboratory, Institute of Biological Sciences, Federal University of Para, Pará, Brazil; ^2^Department of Cell Biology, University of Miami, Miami, FL, United States; ^3^Virus Laboratory, Institute of Biological Sciences, Federal University of Para, Pará, Brazil

**Keywords:** immuno-epigenetic, biomarkers, infectious disease, host, immune response

The ability of microorganisms to infect and cause disease in higher organisms depends on: (i) the nature of the infecting organism(s), (ii) the route of infection, (iii) virulence factors, which enable long-term survival within the organism, and (iv) the immune defense mechanisms of the host. Molecular epidemiological investigations provide evidence regarding the etiology and mechanisms by which microorganisms cause disease, and the results are used to develop strategies for disease prevention. While classic immunogenetic biomarkers (single nucleotide polymorphisms—SNPs) have been shown to be related to resistance or susceptibility to infectious disease, new studies have highlighted alterations in the epigenetic landscape of immune cells as an equally important means of detecting and understanding the progression of infectious diseases. The constant evolutive race between host and infectious agents creates diverse scenarios ranging from differential susceptibility to infection to a large variability at pathogenesis, disease progression, and response to therapy.

In the center of these challenging diseases is the plasticity of the immune response, along with a myriad of environmental factors. This cross-talk among genes, pathways, and environment, that is carried out mainly by epigenetic actors can be used as biomarkers to predict disease progression and prognosis.

In the present Research Topic, original articles and reviews provided a comprehensive overview of immune and epigenetic biomarkers related to infectious diseases.

Bacterial and viral diseases were approached by the original article from Barletta-Naveca et al. who reported an association of Toll-like receptor 1 polymorphism with multibacillary/paucibacillary tuberculosis, along with sociodemographic and behavioral factors, as well as a classic and novel association study of *Chlamydia trachomatis* and *C. pneumoniae* infections, IL-6 and IL-8 polymorphisms, and heart diseases (Almeida et al.). Moreover, Pereira et al. showed that polymorphisms in the FOXP3 gene regulatory region are associated with viral load and liver enzyme levels in chronic viral hepatitis, and Brites-Alves et al. reported that levels of IL-6 are related to the risk of cardiovascular events in HIV-1-infected patients under antiretroviral therapy.

The plasticity of host-pathogen interaction was addressed by Ramsuran et al., who reviewed the polymorphisms in untranslated genomic regions and their role in the regulatory processes of infectious diseases. In a similar, but more focused approach, Ellwanger et al. reviewed the role of SNP in microRNA genes and/or their binding sites in infection by five major human viral pathogens: hepatitis B virus (HBV), hepatitis C virus (HCV), human immunodeficiency virus (HIV), Epstein-Barr virus (EBV), and human papillomavirus (HPV), showing the potential clinical applications of this approach.

Protozoal pathogens were reviewed and highlighted epigenetic processes that occur during *Leishmania* infection, involving histone modifications and non-coding RNAs that modulate the host response to infection. Therapeutic aspects of epigenetics and their possible usefulness as biomarkers were also addressed (Afrin et al.). A systematic review performed by de Aguiar et al. on major epigenetic alterations in arboviruses, predominantly in Dengue, showed the role of microRNA and DNA methylation in secondary Dengue fever.

Finally, two original articles on epigenetic biomarkers showed that hsa-mir-125a-p may be a novel biomarker of liver damage in HBV patients (Coppola et al.), and the role of interference of *Brucella abortus* with microRNA expression affecting the expression of TNF alpha, IL-10, and gualynate-binding protein 5 in host macrophages (Corsetti et al.).

## Concluding Remarks

Epigenetics is commonly defined as gene expression that is inherited but is not dependent on nucleic acid changes. The manuscripts in the present collection focus on some examples of highly important infectious diseases, which are usually associated with immune disorders leading to inflammation. The information presented is interesting and may introduce some new biomarkers of infection, disease progression, and prognosis. Despite our limited knowledge, molecular biology has become a fascinating tool to help unveil the mechanisms explaining the pathology of infection that is mediated by our immunological response. The increasing amount of information generated, which is clearly shown in each manuscript, may seem like unrelated data, but all data are useful, and bioinformatics will soon be able to cope with the enormous amount of information and will be used to investigate the currently available data and direct future studies.

## Author Contributions

All authors listed have made a substantial, direct and intellectual contribution to the work, and approved it for publication.

### Conflict of Interest

The authors declare that the research was conducted in the absence of any commercial or financial relationships that could be construed as a potential conflict of interest.

